# Impact of sinus surgery in people with cystic fibrosis and chronic rhinosinusitis in the era of highly effective modulator therapy: Protocol for a prospective observational study

**DOI:** 10.1371/journal.pone.0310986

**Published:** 2024-09-26

**Authors:** Christine M. Liu, Jakob L. Fischer, Jeremiah A. Alt, Todd E. Bodner, Naweed I. Chowdhury, Anne E. Getz, Peter H. Hwang, Adam J. Kimple, Jess C. Mace, Timothy L. Smith, Zachary M. Soler, Christopher H. Goss, Jennifer L. Taylor-Cousar, Milene T. Saavedra, Daniel M. Beswick

**Affiliations:** 1 Department of Otolaryngology-Head and Neck Surgery, University of California, Los Angeles, Los Angeles, CA, United States of America; 2 Department of Otolaryngology-Head and Neck Surgery, University of Utah, Salt Lake City, UT, United States of America; 3 Department of Psychology, Portland State University, Portland, OR, United States of America; 4 Department of Otolaryngology-Head and Neck Surgery, Vanderbilt Health, Nashville, TN, United States of America; 5 Department of Otolaryngology-Head and Neck Surgery, University of Colorado, Boulder, CO, United States of America; 6 Department of Otolaryngology-Head and Neck Surgery, Stanford University, Stanford, CA, United States of America; 7 Department of Otolaryngology-Head and Neck Surgery, University of North Carolina, Chapel Hill, NC, United States of America; 8 Department of Otolaryngology-Head and Neck Surgery, Oregon Health & Science University, Portland, OR, United States of America; 9 Department of Otolaryngology-Head and Neck Surgery, Medical University of South Carolina, Charleston, SC, United States of America; 10 Department of Medicine, Division of Pulmonary, Critical Care and Sleep Medicine, University of Washington, Seattle, WA, United States of America; 11 Department of Medicine, National Jewish Health, Denver, CO, United States of America; 12 Department of Pediatrics, National Jewish Health, Denver, CO, United States of America; AUSL della Romagna, ITALY

## Abstract

**Introduction:**

Cystic fibrosis (CF) is commonly complicated by chronic rhinosinusitis (CRS). Despite highly effective management options, CRS in people with CF (PwCF+CRS) may be refractory to medical therapy, eventually requiring endoscopic sinus surgery. The impact of sinus surgery on pulmonary, quality of life (QOL), and other outcomes in PwCF+CRS in the expanding era of highly effective modulator therapy has not been fully elucidated. This study aims to determine if endoscopic sinus surgery can offer superior outcomes for PwCF+CRS when compared to continued medical treatment of CRS.

**Methods and analysis:**

This multi-institutional, observational, prospective cohort study will enroll 150 adults with PwCF+CRS across nine US CF Centers who failed initial medical therapy for CRS and elected to pursue either endoscopic sinus surgery or continue medical treatment. To determine if sinus surgery outperforms continued medical therapy in different outcomes, we will assess changes in pulmonary, CF-specific QOL, CRS-specific QOL, sleep quality, depression, headache, cognition, olfaction, productivity loss, and health utility value after treatment. The influence of highly effective modulator therapy on these outcomes will also be evaluated. This study will provide crucial insights into the impact of endoscopic sinus surgery for PwCF+CRS and aid with development of future treatment pathways and guidelines.

**Ethics and dissemination:**

This study has been approved by each institution’s internal review board, and study enrollment began August 2019. Results will be disseminated in conferences and peer-reviewed journals.

**Trial registration:**

This study was registered on ClinicalTrials.gov (NCT04469439).

## Introduction

Cystic fibrosis (CF) is a systemic disease with thick secretions affecting multiple organs, resulting in substantial health and economic burdens [1]. The leading cause of mortality in PwCF is pulmonary dysfunction, often due to secondary bacterial colonization from defective mucociliary clearance [2]. The unified airway theory suggests that similar infectious, inflammatory processes occur in both upper and lower airways, and upper airway infections may contribute to lower airway disease [1]. Most people with CF develop chronic rhinosinusitis (PwCF+CRS) from their inability to clear thickened secretions from the upper airways and paranasal sinuses, and most PwCF will demonstrate clinical or radiographic signs of sinonasal inflammation [3]. The Cystic Fibrosis Foundation (CFF) patient registry indicates that over half of adults report symptomatic sinus disease [4].

**Fig 1:**
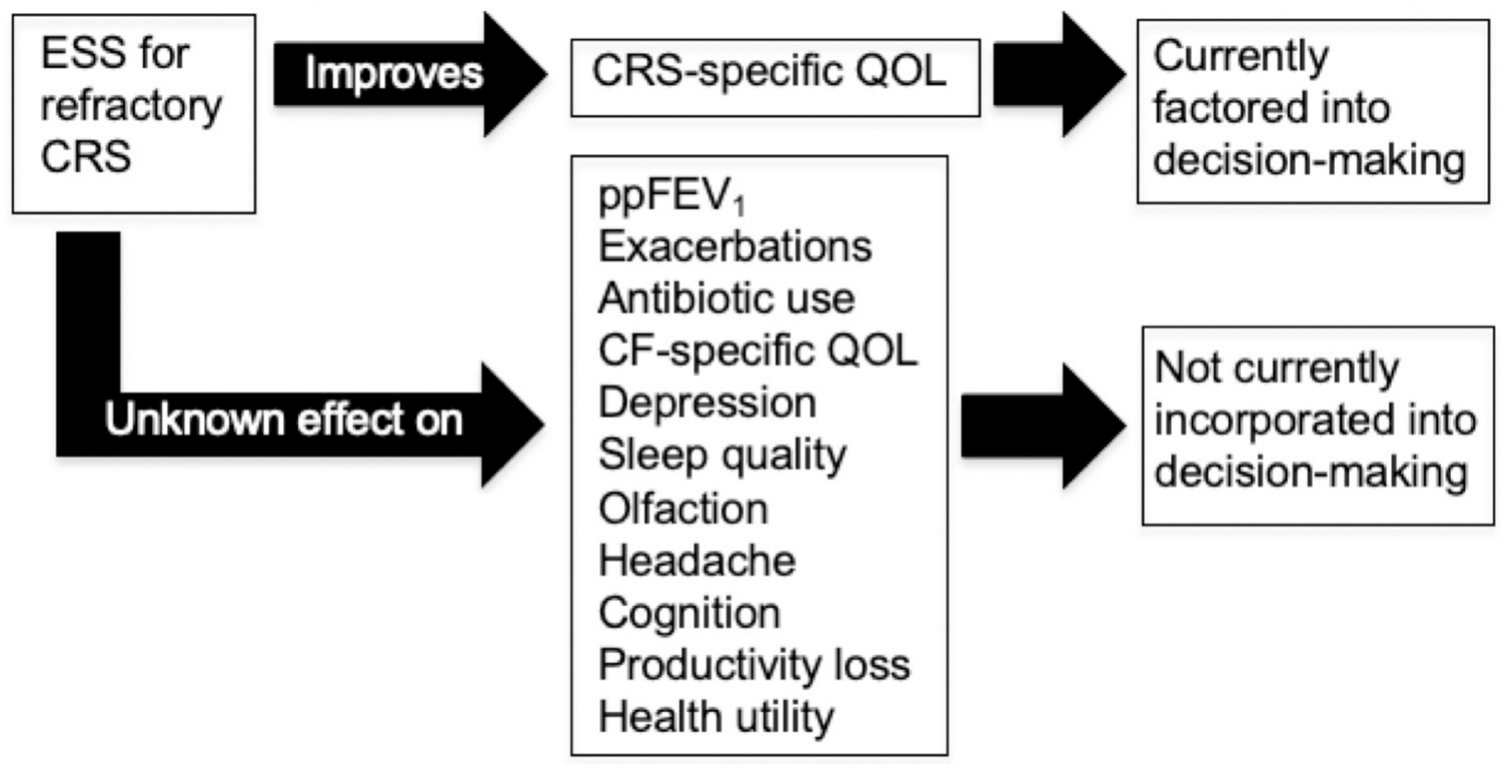
Unaccounted factors in endoscopic sinus surgery decision-making for people with cystic fibrosis. ESS, endoscopic sinus surgery; CMT, continued medical therapy; PwCF+CRS, people with cystic fibrosis and comorbid chronic rhinosinusitis; CF, cystic fibrosis, CRS, chronic rhinosinusitis; QOL, quality of life; ppFEV_1_, percent of predicted expiratory volume in the first second.

Endoscopic sinus surgery (ESS) is a treatment option for PwCF+CRS that serves to enlarge sinus openings, clear inspissated mucus, and facilitate application of topical therapies. Based on consensus guidelines, ESS is recommended for adults and children with CF with symptomatic CRS refractory to appropriate medical therapy [5, 6]. While ESS improves sinonasal symptoms and CRS-specific quality of life in PwCF+CRS [7], there is limited data evaluating ESS treatment outcomes in the era of highly effective modulator therapy (HEMT) and potential benefits of ESS in regions beyond the paranasal sinuses (**Fig 1**).

The CF treatment landscape is rapidly transforming with the introduction of CF transmembrane conductance regulator (CFTR) modulator therapies. The triple combination therapy elexacaftor/tezacaftor/ivacaftor (ETI) is clinically available in the United States (US) for ~90% of people with CF > 2 years of age based on variant status and considered a HEMT [8]. ETI therapy has been associated with improved sinus computed tomography (CT) scan scores and CRS symptom severity, but does not fully resolve the symptoms of CRS in adults even with extended therapy for up to two years [9, 10]. While ETI has demonstrated high treatment efficacy, approximately 10% of people in the US are not eligible based on variant status and still others may not be able to tolerate the medication due to potential side effects [11]. Understanding the impacts of ESS in the modern era, while accounting for the effect of ETI, remains critical for the CF community.

Prior research on pulmonary outcomes after ESS has produced mixed results [12, 13], and has mainly consisted of small, retrospective studies. The current research landscape on ESS in PwCF is largely limited to retrospective case-control studies, constrained by patient numbers and the challenges of randomizing surgical interventions. This prospective, multi-institutional cohort-study aims to comprehensively determine the impact of ESS on PwCF+CRS in the era of highly effective modulator therapy.

## Materials and methods

### Study design, eligibility, and recruitment

This is a multi-institutional prospective, observational cohort study of patients electing to undergo either ESS or continued medical therapy (CMT) for CF-related CRS after having failed initial medical therapy. Patients will be enrolled at one of nine participating CF centers across the US.

Potential participants will be considered for study inclusion if they are adults (age 18–99 years), diagnosed with CF by genetic testing and/or sweat chloride testing, and have persistent CRS based on multi-disciplinary guidelines [5, 6]. Patients who underwent ESS in the 12 months preceding study enrollment, plan to have follow-up care at a non-participating institution, are unable to complete post-operative outcome measures, or started or modified new CFTR modulator therapy within 90 days of study enrollment will be excluded **(Table 1).** Participants consenting to study will be included in one of two study groups: those who elect to undergo ESS and those who pursue CMT. Extent of surgery, including use of extended approaches, will be tracked and analyzed.

**Table 1:** Inclusion and exclusion criteria.

Inclusion Criteria	Exclusion Criteria
Age 18 years or older	Underwent ESS in past 12 months
Diagnosed with CF as established by genetic testing combined with clinical assessment and/or sweat chloride	Will obtain follow up care at nonparticipating institutions
Diagnosed with CRS by multidisciplinary sinusitis guidelines	Unable to complete outcome measures
CRS symptoms persisting beyond initial medical treatment	Started new CFTR modulator therapy less than 90 days prior to screening or anticipated to begin during study period

ESS, endoscopic sinus surgery; CF, cystic fibrosis; CRS, chronic rhinosinusitis, CFTR, cystic fibrosis transmembrane receptor

All participants will be recruited from otolaryngology clinics at one of nine US institutions that are also CF Centers. Recruitment will occur at The University of California, Los Angeles (UCLA; Los Angeles, CA); University of Colorado (Aurora, CO); National Jewish Health (Denver, CO) including its affiliate Saint Joseph’s Hospital (Denver, CO); the University of Utah (Salt Lake City, UT); the Oregon Health and Science University (Portland, OR); the Medical University of South Carolina (Charleston, SC); the University of North Carolina (Chapel Hill, NC); Vanderbilt University Medical Center (Nashville, TN); and Stanford University (Palo Alto, CA); with UCLA as the coordinating site. Our recruitment goal is 150 patients. Recruitment techniques include routine face-to-face encounters, routine telephone encounters, and referrals from outside medical treatment facilities. An investigator or site coordinators using documents approved by each institution’s internal review board using Good Clinical Practice guidelines [14] will obtain written informed consent for all study participants.

### Objectives & endpoints

- Objective 1: Determine whether ESS outperforms CMT for CF-related CRS over 12 months follow-up using endpoints of percent of predicted expiratory volume in the first second (ppFEV_1_), the rate of pulmonary exacerbations (PEx), and antibiotic utilization for exacerbations.
- Objective 2: Delineate changes in QOL and sinus-related comorbidities for participants who undergo ESS through 12 months of follow-up compared to CMT. Endpoints include assessing the impact of treatment on CF-specific QOL, CRS-specific QOL, sleep quality, depression, headache, cognition, and olfaction, which will be assessed using validated patient-reported outcome measures.
- Objective 3: Define changes in productivity loss (PL) and health utility value (HUV) in PwCF+CRS who undergo ESS through 12 months follow-up compared to standard CMT. Endpoints are validated patient-reported outcome measures to quantify PL and HUV.

All objectives will also evaluate the impact of ESS compared to CMT on pulmonary, rhinologic, QOL outcomes accounting for HEMT. Objectives and endpoints are listed in **Table 2**.

**Table 2:** Study objectives and endpoints in prospective observation study in adults with cystic fibrosis and comorbid chronic rhinosinusitis.

Objective	Endpoint	Assessments
1) Determine whether ESS outperforms CMT for CF-related CRS using measures in pulmonary function, the rate of PEx, and antibiotic use in over 12 months follow-up	Change in percent of predicted expiratory volume in the first second (ppFEV_1_) at initial visit and months 3,6,9, and 12.	NA
	Rate of PEx, and antibiotic utilization for exacerbations compared between treatment groups.	
2) Delineate changes in CF-specific quality of life and sinus-related comorbidities for subjects who undergo ESS through 12 months of follow-up and assess if ESS outperforms CMT in these areas.	Descriptive summary of CF-specific QOL, CRS-specific QOL, sleep quality, depression, headache, cognition, and olfaction; and improvement in scores and comparisons between groups from baseline through 12 months post-treatment.	CFQ-R (CF QOL), SNOT-22 (CRS QOL), PSQI (Sleep), PHQ-9-revised (Depression), HIT-6 (Headache), CFQ (Cognitive function), QOD (Olfaction), SIT (Smell Identification Test)
3) Delineate changes in productivity loss and health utility value for subjects who undergo ESS through 12 months of follow-up and evaluate if ESS outperforms CMT in these areas.	Descriptive summary of productivity and health utility value; and improvement in scores and comparisons between groups from baseline through 12 months post-treatment.	WPAI (Productivity), EQ-5D (Health Utility Value)

ESS, endoscopic sinus surgery; CMT, continued medical therapy; CF, cystic fibrosis, QOL, quality of life; ppFEV_1_, percent of predicted expiratory volume in the first second; PEx, pulmonary exacerbation; QOL, quality of life; CFQ-R, Cystic Fibrosis Questionnaire-Revised; SNOT-22, 22-question Sino-Nasal Outcome Test; PHQ-9-revised, Patient Health Questionnaire-9-revised; PSQI, Pittsburgh Sleep Quality Index; CFQ, Cognitive Failures Questionnaire; HIT-6, Headache Impact Test; QOD, Questionnaire of Olfactory Disorders; SIT, Smell Identification Test; WPAI, Work Productivity and Activity Impairment-Specific Health Problem; HUV, EQ-5D, 5-dimensional EuroQol questionnaire.

### Evaluation timeline

The following information will be collected at the patient’s initial consultation visit and 3, 6, 9, and 12 months follow-up with +/- 4-week ranges for follow up time points. These time points were selected based on the prior frequency that people with CF had routine clinic visits before the regulatory approval of ETI, as the study was designed in 2018.

- Pulmonary function testing data that was obtained as clinical standard of care based on clinical records to analyze ppFEV1.
- PEx-related medical history and diagnostic testing; and data regarding utilization of oral and IV antibiotic utilization
- Questionnaires: Cystic Fibrosis Questionnaire-Revised (CFQ-R), 22-question Sino-Nasal Outcome Test (SNOT-22; © Washington University, St. Louis, MO), Pittsburgh Sleep Quality Index (PSQI), Patient Health Questionnaire-9-revised (PHQ-9-revised), Cognitive Failures Questionnaire (CFQ), and Headache Impact Test (HIT-6)
- Evaluations for olfactory function: 40-question Smell Identification Test (SIT; © Sensonics International, Haddon Heights, NJ) and the Questionnaire of Olfactory Disorders (QOD) for olfactory specific QOL assessment
- Productivity loss and health utility data: Work Productivity and Activity Impairment-Specific Health Problem (WPAI) and 5-dimensional EuroQol questionnaire (EQ-5D-5L; © EuroQol Group, Rotterdam, The Netherlands) to quantify health utility value.

- Mucus and nasal microbiome samples

A set of questions on attitudes regarding sinusitis will be completed once at the baseline visit. Data collection time points are identical for both ESS and CMT subjects and are further detailed in **Table 3.**

**Table 3:** Data collection time points in months.

Data collection time points	Baseline	3	6	9	12
History and physical, computed tomography	x				
Disease and socio-demographic factors	x				
Nasal endoscopy	x		x		
Treatment option: ESS vs. CMT	x				
Medication, CFTR modulator, cross-over data	x	x	x	x	x
PFTs, PEx data, antibiotic use	x	x	x	x	x
CFQ-R, SNOT-22, QOD, PSQI, PHQ-9-revised, CFQ, SIT, HIT-6, WPAI, HUV	x	x	x	x	x
Nasal mucus sponge, nasal microbiome swab	x	x	x	x	x
Blood, excised ethmoid sinus tissue*	x				

ESS, endoscopic sinus surgery; CMT, continued medical therapy; PwCF+CRS, people with cystic fibrosis and comorbid chronic rhinosinusitis; CF, cystic fibrosis, QOL, quality of life; CFTR, cystic fibrosis transmembrane receptor; PFT, pulmonary function test; PEx, pulmonary exacerbation; CFQ-R, Cystic Fibrosis Questionnaire-Revised; SNOT-22, 22-question Sino-Nasal Outcome Test; PHQ-9-revised, Patient Health Questionnaire-9-revised; PSQI, Pittsburgh Sleep Quality Index; CFQ, Cognitive Failures Questionnaire; HIT-6, Headache Impact Test; QOD, Questionnaire of Olfactory Disorders; SIT, Smell Identification Test; WPAI, Work Productivity and Activity Impairment-Specific Health Problem; HUV, health utility value; EQ-5D, 5-dimensional EuroQol questionnaire.

*These are for surgical cases only. Centers which routinely perform blood draws during clinic visits may also collect blood specimens at multiple follow up visits.

### Baseline measures

All participants will undergo baseline history and physical examination, CT, and nasal endoscopy. Key disease factors and potential predictors will also be obtained at initial visit with rhinologist. These include: age, gender, race/ethnicity, BMI, age at CF diagnosis, genotype, ppFEV1, CFTR modulator use details, prior ESS history, pancreatic insufficiency, *Pseudomonas* positivity, nontuberculous mycobacteria infection, lung transplantation status, insurance status, income, occupation, education, enrollment site, and medication use data. To measure CRS severity at baseline, we will use endoscopic and radiographic techniques. CT images will be scored via the Lund-Mackay system [15], and diagnostic nasal endoscopy will be performed and scored according to the Lund-Kennedy scoring system [16].

### Outcome assessments

To address objective 1, primary pulmonary outcome measure will be ppFEV_1_. Absolute values of FEV1 and FVC will be assessed as secondary spirometry measures, enabling evaluations of the FEV1/FVC ratio [17]. Forced expiratory flow at mid-lung volume (FEF25-75) is an important marker of small airway disease and will also be evaluated [18]. Secondary outcomes include the frequency of PEx (both outpatient and inpatient) and intravenous (IV) and oral antibiotic use. PEx will be defined by the Rosenfeld criteria [19]. The number of PEx, number of hospitalizations, and days of IV and oral antibiotics used will be tracked.

To address objective 2, quality of life outcome measures will include CFQ-R, evaluating CF-specific QOL [20], and the SNOT-22, evaluating QOL in subjects with CRS [21]. PHQ-9-revised will be used for depression screening [22], and the PSQI will measure sleep duration and quality [23]. CFQ will be used for cognitive assessment [24]. The HIT-6 will be utilized to determine headache severity [25]. For evaluation of olfactory function, we will use patient-reported and objective olfactory function tests. The QOD will assess the impact of olfactory dysfunction [26]. The SIT is a measure of objective olfactory function and complements the patient-reported QOD survey [27].

To address productivity loss and health utility in objective 3, the WPAI will be used to assess the impact of a specific health problem on normal work/school or daily activity [28]. Health utility value (HUV) will be quantified from the EQ-5D [29].

### Biospecimen collection

Biospecimens will be collected to assess nasal microbiology, inflammatory cytokines, and tissue histology, and analysis will investigate associations between biological findings and clinical outcomes. For both ESS and CMT groups, microbiome samples from the middle meatus will be obtained endoscopically. Nasal mucus specimens will be collected using sponges placed in the olfactory cleft for inflammatory cytokine analysis in both treatment groups.

For ESS patients, blood and excised tissue that would normally be removed during surgery from the ethmoid sinus will be collected at surgery. The tissue will be stored in a vial with RNAlater (Sigma Aldrich, MilliporeSigma, St. Louis, MO). Blood will be drawn during surgery using standard venipuncture techniques and collected in PAXGene Blood RNA tubes (Becton & Dickinson, Franklin Lakes, NJ). Centers which routinely perform blood draws during clinic visits may also collect blood specimens at multiple visits. All specimens will be collected at enrolling centers and will be transferred to one center for batch analysis to minimize variability.

### Statistical methods

For all objectives, we will verify predictive model assumptions and test goodness-of-fit, considering alternative models if needed. We will account for potential site-specific differences as fixed effects in our models. Other methods to address site clustering include mixed effects models and generalized estimating equations.

For Objective 1, mixed effects model will be used to assess the impact of ESS versus CMT on ppFEV_1_ changes at initial and subsequent visits. Fixed effects will include baseline FEV1 (FEV1 > 80% predicted, <80% and > 50%, and <50%), CF variant status, use of CFTR modulator therapy, sex, prior sinus surgery, time and treatment; patients will be treated as random effects. An unstructured variance approach will model within-subject errors, and we will explore other covariance if needed. As exploratory analysis, we will compare ppFEV_1_ from a year before enrollment to post-treatment values using the same model. Using a generalized linear model with a Poisson distribution and prior year data, we will assess the impact of ESS versus CMT on exacerbation rates and antibiotic use, complemented by descriptive statistics for sociodemographic factors.

For Objective 2, we will describe patient characteristics using summary statistics. Treatment effects on patient reported-outcome measures (CFQ-R, SNOT-22, PHQ-9, PSQI, CFQ, QOD, and SIT) will be explored via a mixed effects model, adjusting for multiple comparisons using a gate-keeping procedure and assessing for self-report bias in patient-reported outcomes by comparing them to FEV1.

For Objective 3, we will correlate productivity costs with patient demographics, disease factors, pulmonary status, and QOL impairment. We will use a mixed effects model to evaluate these outcomes.

Additionally, we will implement multiple approaches to compare changes in longitudinal treatment outcomes listed in objectives 1–3 between the ESS and CMT groups. First, multivariate Profile Analysis (MPA) [30], unlike the mixed model approach, can handle multiple variables simultaneously and does not require stringent assumptions. Two main effects from the MPA model are of interest: 1) Tests on patient group differences at baseline on the outcomes in the study and 2) tests of “parallelism”, determining if the outcome trajectories (or profiles) change similarly in the two patient groups over time. Second, we will apply Propensity Score Matching (PSM) to test for the causal effects of ESS and CMT on study outcomes, and to create balanced treatment comparisons to address self-selection biases [31, 32]. Other methods using propensity scores like weighting or stratification, and instrumental variable (IV) approach, will also be considered to adjust for potential biases and estimate causal effects.

Consideration was given to restricting enrollment to surgically naïve patients; however, this approach would limit enrollment. We will therefore explore subgroup analyses in surgically naïve study participants and sensitivity analyses to look at changes over time in outcomes. We also plan to account for surgical history in a mixed effects model that includes previous sinus surgery.

### Statistical power and sample size

Sample size and power were calculated using software G Power (v. 3.1). Type I error will be controlled at two-sided 5% level. Sample size calculations were initially based on our primary QOL outcome measure (SNOT-22 scores) for objective 2, given strong evidence of improvements in the literature [33]. This was then applied to objective 1. For objective 2, detecting SNOT-22 score changes pre- and post-12 months ESS requires 9 patients for 80% power. To determine changes between treatment groups over time, considering a potential 30% cross-over between ESS and CMT groups, 106 patients are needed for 80% power, as indicated by prior data [34]. Accounting for 20% potential dropouts, our recruitment target is 150 participants, with an anticipated enrollment of approximately 170 across all centers.

Regarding ESS effects on ppFEV_1_, previous mixed results complicate clear expectations to use in power calculations for objective 1. Assuming 170 participants (factoring in 20% dropout), we will have 80% power to detect a 1.6% ppFEV_1_ change for objective 1 and a 2.3% ppFEV_1_ change for objective 1 accounting for HEMT.

### Patient retention and missing data analysis

Missing data will be presumed to be missing at random (MAR). For our primary analyses, we will use mixed effects model to handle missing data issues based on MAR using Full Information Maximum Likelihood (FIML) estimation.

### Data management

Study data will be managed using REDCap, a secure application offering user-friendly forms, real-time data entry validation, and de-identified data export features. The database is hosted at UCLA Health for centralized data handling with secure, electronic access provided to all external study teams.

### Patient and public involvement

This study engaged PwCF+CRS from the initial design stage. Through focus group interviews, we gleaned PwCF’s preferences regarding post-ESS outcomes, ensuring that our research aims aligned with their priorities. This feedback directly shaped our research questions and outcome measures of pulmonary function, olfaction, frequency of PEx, and QOL. When PwCF+CRS were queried about potential study design options, individuals were much more likely to participate in an observational study than a trial randomized to ESS or CMT, leading to the selection of an observational study design. We aim to publish study results with CF Foundation’s community members, healthcare professionals, and CF researchers.

### Ethics and dissemination

This study was registered on ClinicalTrials.gov (NCT04469439) and conducted in compliance with the Declaration of Helsinki and International Conference on Harmonization and Good Clinical Practice guidelines and applicable regulatory requirements. Each participating site received approval from their local Institutional Review Board (IRB). IRB approval for the primary coordinating site was granted by UCLA approval number 20–002079.

The results of this study will be disseminated during national and international scientific conferences and published in peer-reviewed journals. Stakeholders will be informed via CF Foundation’s research newsletters.

## Current status

This study started in August 2019 and as of October 2023, we have 87 enrolled participants. We are continuing to enroll at all sites.

## Discussion

In this multi-institutional prospective study, our primary objective is to evaluate post-ESS outcomes in PwCF+CRS in the era of highly effective modulator therapy. We will examine pulmonary function, PEx rates, antibiotic use, QOL, productivity loss, and health utility value over a 12-month period post-ESS. Our study uniquely will compare the impact of HEMT on outcomes between PwCF+CRS who undergo ESS and those opting for CMT.

Our study leverages a prospective, multi-institutional design, mitigating the limitations of small sample size and common biases associated with retrospective review. We acknowledge the limitations of an observational design instead of a randomized controlled clinical trial (RCT). This design was selected based on challenges of randomizing surgical interventions and PwCF’s reservations about randomization to ESS or delayed intervention [35]. Many PwCF expressed reluctance to be randomized, which would likely have led to enrollment difficulties in a RCT [35]. Further, there are notable difficulties in randomizing study participants to surgical interventions that require general anesthesia. These include the high cost of interventions that the study may have to fund (ESS estimated at > $10,000 USD/case) [36] and ethical considerations, such as employment of sham surgery in a placebo control arm [37]. To minimize potential bias, we will employ advanced statistical techniques like MPA and propensity score matching. Our method is poised to produce robust data, including endpoints that can provide some of the first insights of CRS outcomes in the current era. Data accumulated from this study will provide preliminary data upon which to design future trials.

In contrast to prior post-ESS studies evaluating ppFEV_1_ and demonstrating mixed results [12, 13], our research will thoroughly examine the relationship between ESS and pulmonary function in PwCF by assessing not only spirometry, but also PEx and antibiotic use, all crucial outcomes due to their significant impact on morbidity, QOL, and associated costs [36]. Previous findings from the period before widely available HEMT indicate that people without CF who undergo ESS have improved sinonasal symptoms and CRS-specific QOL [7, 37]. However, this study will comprehensively evaluate in the HEMT era the impact of ESS on multiple validated metrics such as CF-specific QOL, CRS-specific QOL, sleep, depression, olfaction, and cognition. Similar to non-CF individuals with CRS, PwCF suffer from depression [38], poor sleep [39], headaches [40], impaired olfaction [41], and cognitive limitations [42]. Prior work has demonstrated improvement in these comorbid conditions after ESS in non-CF+CRS individuals [43–46]; however, depression, sleep, and olfactory outcomes post-ESS require further study in PwCF who have systemic disease and a vastly different burden than people with CRS without CF.

Annual productivity loss per person with CF is estimated at $15,935 [47], compared to non-CF, refractory CRS patients with PL of $10,077 [48, 49]. CRS patients have demonstrated diminished work performance or disease-related absenteeism that may be magnified in PwCF who may require daily routine treatments [48], for those not on HEMT, or who require additional care related to PEx [50]. Prior research has shown that ESS is associated with improvements in PL in non-CF+CRS patients [51], and this is an area meriting investigation in PwCF+CRS. HUV is a measure of generalized QOL that has been used for the valuation and comparison of treatment in CRS and CF [52, 53]. The study of HUV in CF has primarily focused on lung transplantations and management of PEx [52]. With the exception of PwCF with no exacerbations, CF patients have lower mean HUV compared to the general US population (0.85 [SD 0.81]) and non-CF CRS population (0.81[SD 0.13]) [53]. In non-CF+CRS patients, prior research has indicated clinically relevant improvements in HUV post-ESS, unlike those on CMT [54]. Other prior work has shown that HEMT leads to clinically relevant improvements in HUV [55, 56]. Our study seeks to explore ESS’s impact on HUV for PwCF+CRS and will assess if HUV improvement occurs on top of that previously removed with HEMT.

Our proposed study extends beyond pulmonary function to encompass QOL, PL, and HUV outcomes, reflecting the evolving landscape of cystic fibrosis management as lifespans continue to lengthen with expanding use of HEMT [57]. By exploring the interplay between post-ESS outcomes and HEMT, we aim to better understand treatment outcomes and optimize management of CF-related CRS. We also plan to characterize olfactory dysfunction’s impact on eating patterns and nutritional status, which hold significant implications for pulmonary function in PwCF [58]. The results from this study will serve as valuable preliminary insights for designing future trials and refining treatment strategies.

In conclusion, investigating pulmonary function, QOL, and health economic outcomes related to CF-CRS presents a significant opportunity to expand understanding of this disease complication. The proposed study will bring together participants across multiple CF centers to garner a large sample size. We anticipate uncovering valuable insights into the optimal management of CF-CRS. Several questions remain unanswered, such as the long-term effects of ESS and HEMT, particularly in relation to depression, sleep, and olfactory outcomes in PwCF. Future research should delve further into these areas, providing a more nuanced understanding of the multifaceted impact of ESS in PwCF+CRS.

## Supporting information

S1 Checklist STROBE checklist for observational cohort study. List of essential information to include when reporting results from an observational cohort study based on STROBE (STrengthening the Reporting of OBservational studies in Epidemiology) guidelines.
(DOC)
